# Medical Education

**Published:** 1874-11-15

**Authors:** 


### MEDICAL EDUCATION.

THE editor of the *Philadelphia Medical Times*, in a recent number of his journal, commenting on this subject, very justly places a large part of the responsibility for the continuance of the exceedingly defective system of medical college instruction, adopted by all except two or three of the colleges in this country, directly on the old and well-patronized schools of his own city. After complimenting the ability of their respective faculties, their extensive clinical advantages, &c., he says: "It is not of these things we complain, but candor forces us to acknowledge with sorrow, that any student who does not want so much to learn as to get the title of M.D., and the right to practice, will find in the Philadelphia diplomas the maximum of respectability, with almost a *minimum of necessary attainments*." This is nothing more than the exact truth. The medical schools in Philadelphia and New York, with all the prestige of age, and the patronage of large classes, not only plod on with a short annual course of ungraded, repetitional medical instruction, but they really pay less attention, both to the rule requiring three years of study and to the actual attainments of the candidates for graduation, than three-fourths of the so-called smaller schools in other parts of the country. If the medical colleges in those two cities, would, at the opening of their next annual sessions, grade their curriculum into three consecutive courses, one adapted to each year of study; grade their classes accordingly; enforce rigid examinations at the close of each term on the branches taught in each division, as recommended by the College Conventions of 1867 and 1870, every respectable medical college in the whole country would follow their example within three years, and our system of medical education would be no longer ridiculed as a sham and a national disgrace. How much longer will the honorable members of the respective faculties of those schools willingly bear the responsibility of continuing a system that annually collects in their halls, classes composed of all grades of students mixed, and, in sixteen or eighteen weeks, pours into their ears, in heterogeneous confusion, oral instruction in all the departments of the science and art of medicine, and call it *education?*

Will those responsible for the management of each of the medical schools in Philadelphia and New York consider carefully the hint given to the oldest of them in the following paragraph in the editorial of the *Medical Times?* "Since the University of Pennsylvania has been the foster-mother of the present system, would that it had celebrated its centennial anniversary by stepping up to a higher and nobler plane! It has lost, however, the honor of being the first institution to reform; *if it hesitates much longer* it may have the *shame of being among the last*."

